# Towards an open and synergistic framework for mapping global land cover

**DOI:** 10.7717/peerj.11877

**Published:** 2021-08-04

**Authors:** Jiyao Zhao, Le Yu, Han Liu, Huabing Huang, Jie Wang, Peng Gong

**Affiliations:** 1 Ministry of Education Key Laboratory for Earth System Modeling, Department of Earth System Science, Tsinghua University, Beijing, China; 2 Ministry of Education Ecological Field Station for East Asia Migratory Birds, Tsinghua University, Beijing, China; 3 School of Geospatial Engineering and Science, Sun Yat-Sen University, Guangzhou, China; 4 State Key Laboratory of Remote Sensing Science, Institute of Remote Sensing and Digital Earth, Chinese Academy of Sciences, Beijing, China; 5 Department of Geography and Department of Earth Sciences, University of Hongkong, Hongkong, China

**Keywords:** Global land cover change, Open and synergy mapping, Land cover series

## Abstract

Global land-cover datasets are key sources of information for understanding the complex inter-actions between human activities and global change. They are also among the most critical variables for climate change studies. Over time, the spatial resolution of land cover maps has increased from the kilometer scale to 10-m scale. Single-type historical land cover datasets, including for forests, water, and impervious surfaces, have also been developed in recent years. In this study, we present an open and synergy framework to produce a global land cover dataset that combines supervised land cover classification and aggregation of existing multiple thematic land cover maps with the Google Earth Engine (GEE) cloud computing platform. On the basis of this method of classification and mosaicking, we derived a global land cover dataset for 6 years over a time span of 25 years. The overall accuracies of the six maps were around 75% and the accuracy for change area detection was over 70%. Our product also showed good similarity with the FAO and existing land cover maps.

## Introduction

Global land cover datasets provide key information for understanding the complex interactions between human activities and global change (Running, 2008). They are also some of the most critical variables for studies of climate change (Bounoua et al., 2002; Hibbard et al., 2010), habitat and biodiversity (Buchanan et al., 2009; Hall et al., 2011), carbon cycling (De Moraes et al., 1998; DeFries et al., 1999; DeFries et al., 1995; Poulter et al., 2011), and public health (Liang et al., 2010; Xu et al., 2004). Land cover change can influence the energy balance and biogeochemical cycles (Claussen, Brovkin & Ganopolski, 2001; DeFries et al., 1999) and it can further affect climate change, surface characteristics and the provision of ecosystem services (Pielke, 2005; Reyers et al., 2009; Zhao, Pitman & Chase, 2001). Therefore, better frequent land cover observations are desirable for understanding global environmental change (Liu et al., 2021).

Global land cover mapping has experienced rapid development in the past decades and the spatial resolution of the global land cover maps has increased from kilometer scale to 10-m scale at its finest (Feng & Li, 2020; Gong et al., 2019). The early global land cover maps, the International Geosphere-Biosphere Program Data and Information System’s land cover dataset (IGBP DISCover) (Loveland & Belward, 1997; Loveland et al., 2000) and the University of Maryland land cover dataset (UMD) (Hansen et al., 2000), were released in the 1990s when the Advanced Very High-Resolution Radiometer (AVHRR) data obtained from the National Oceanic and Atmospheric Administration (NOAA) made it possible to map land cover at large scales. With the availability of satellite data at resolutions finer than AVHRR, global land cover datasets with hectometer resolution were developed in the 2000s; for example, land cover from the Moderate Resolution Imaging Spectroradiometer (MODIS) (Friedl et al., 2002; Friedl et al., 2010). In the past 10 years, abundant satellite data has enabled even more precise land cover mapping. For instance, global land cover maps with 30 m-resolution based on Landsat images (Finer Resolution Observation and Monitoring of Global Land Cover, FROM-GLC) (Gong et al., 2013), and 10-m resolution, based on Sentinel 2 (FROM-GLC10), were developed recently (Gong et al., 2019). However, those finer resolution land cover products are hard to be updated to cover long time series due to low data availability for Landsat in the past (Liu et al., 2021; Yu, Shi & Gong, 2015). By aggregating and fusing Landsat and MODIS images, which have different spatial resolutions and observation frequencies, researchers have made progress on land cover mapping by improving their accuracy (Yu, Wang & Gong, 2013) and increasing their observation frequency very recently (Liu et al., 2021).

The MODIS land cover datasets provided researchers with the first long time series land cover at 250-m resolution (Friedl et al., 2002; Friedl et al., 2010). Many efforts have also focused on generating consistent land cover series from MODIS images (Wang et al., 2015). In 2017, European Space Agency Climate Change Initiative (ESA-CCI) released their annual global land cover series from 1992 to 2015 (extended to 2018 later), with 300 m spatial resolution, making it another long annual global land cover series (ESA, 2017). Land cover series were also released on a country scale, such as China’s Land-Use/cover Datasets (CLUDs) (Liu et al., 2014; Xu et al., 2020), United States Geological Survey (USGS) National Land Cover Database (NLCD) (Yang et al., 2018), the National Dynamic Land Cover Dataset of Australia (Lymburner et al., 2011), and the Decadal Land Use and Land Cover Classifications across India (Roy et al., 2016), etc. Meanwhile, single-type thematic land cover series with higher spatial resolution have been developed, such as global forest change by Hansen et al. (2013), global annual water layer by Pekel et al. (2016) and global impervious surface change by Gong et al. (2020). Those local and thematic datasets provide opportunities to improve general land cover maps with local and thematic knowledge (Gong et al., 2016).

In this study, we put forward an open and synergistic framework for combining supervised classification with training samples and aggregating existing multisource land cover datasets. With this framework, novel global land cover maps with a spatial resolution of 30 m every 5 years from 1990 to 2015 have been developed.

## Materials & Methods

### Dataset used in this study

**Figure 1:**
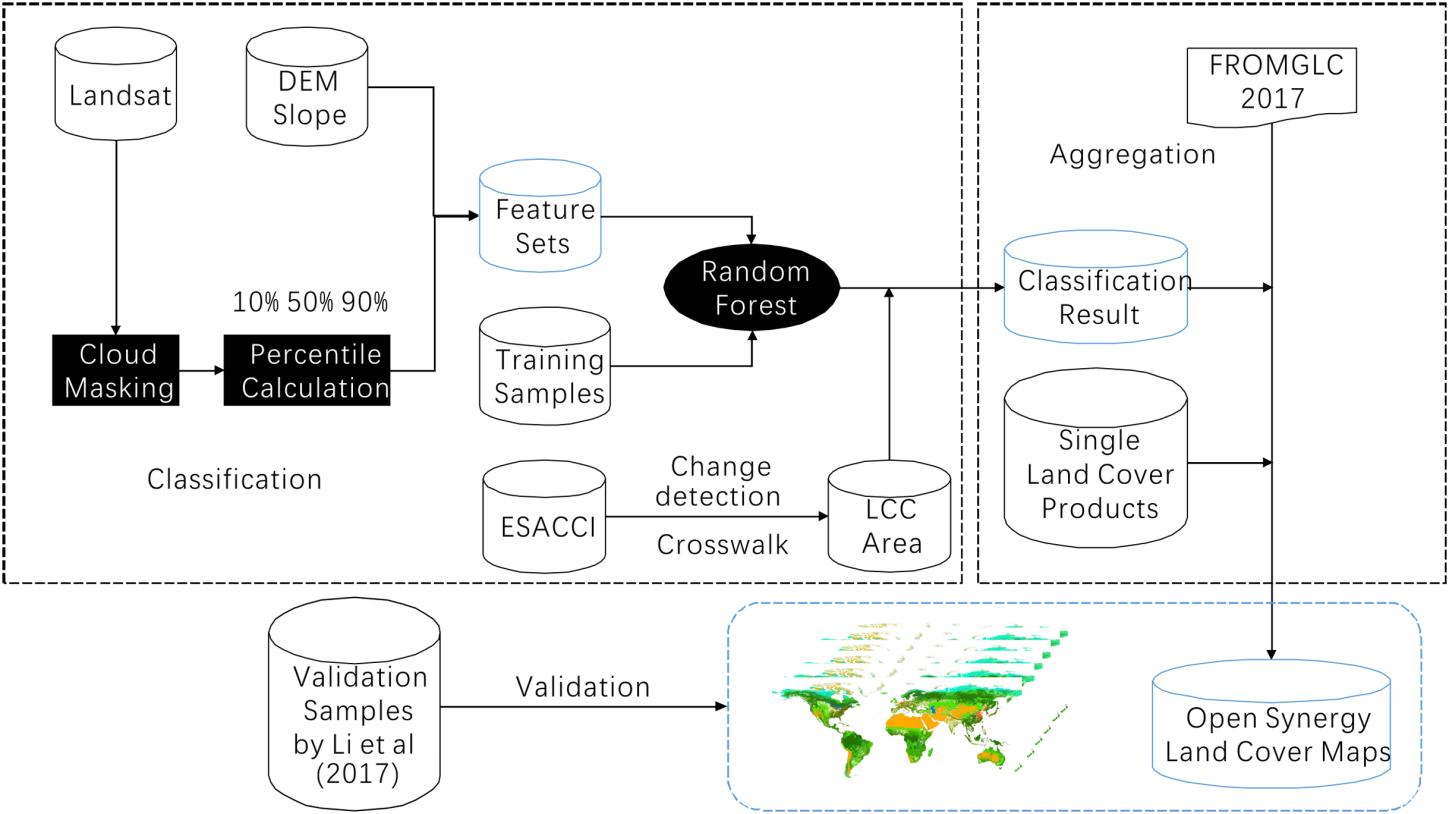
Overall accuracy of the land cover map for 2015 in each ecoregion.

The workflow of this study was shown in Fig. 1. Multiple datasets were used in this study, including the FROM-GLC global land cover map in 2017, which was the most up to date and accurate land cover map among the three FROM-GLC maps in 2010, 2015 and 2017. Landsat surface reflectance dataset, The Shuttle Radar Topography Mission (SRTM) digital elevation model (DEM), ESA-CCI and three recent single-type land cover datasets (see Table 1).

**Table 1:** Summary of multiyear land cover datasets.

Product type	Name	Spatial resolution	Duration	Reference
Global land cover	ESA-CCI	300 m	1992–2018	ESA, 2017
	FROMGLC	30 m	2017	Gong et al., 2013
Impervious	GAIA	30 m	1985–2018	Gong et al., 2020
Forest	GLADForest	30 m	2000–2019	Hansen et al., 2013
Water	JRC_GSW	30 m	1984–2019	Pekel et al., 2016

FROM-GLC at 30-m resolution for 2017 was used as the base map for developing the global open synergistic maps every 5 years (available at: http://data.ess.tsinghua.edu.cn/). The land cover types included cropland, forest, shrubland, water, wetland, tundra, impervious surface and bareland.

In 2017, the ESA released the first time series of annual global land cover maps at 300-m resolution, spanning a 27-year period from 1992 to 2018 and dividing the global land into 22 land cover types (available at: http://maps.elie.ucl.ac.be/CCI/viewer/index.php). This global dataset was made consistent by decoupling the land cover mapping and land cover change detection. The land cover mapping used the full archives of five different satellite missions providing daily observations of the Earth, including NOAA-AVHRR high-resolution picture transmission (HRPT), Systeme Probatoire d’Observation de la Terre (SPOT) Vegetation, ENVIronmental SATellite (ENVISAT)-Medium Resolution Imaging Spectrometer (MERIS) full spatial resolution (FR) and reduced spatial resolution (RR), ENVISAT-Advanced Synthetic Aperture Radar (ASAR), and PRoject for On-Board Autonomy (PROBA)-Vegetation for the most recent years. Because of the long time series and good consistency, we used the ESA-CCI land cover datasets to detect land cover change locations in this study.

The Landsat surface reflectance dataset was used for classifying land cover in the land cover change locations. The Collection 1 Surface Reflectance data are generated using the Land Surface Reflectance Code (LaSRC) (version 1.4.1), which uses the coastal aerosol band to perform aerosol inversion tests, integrates auxiliary climate data from MODIS, and uses a unique radiative transfer model (Vermote et al., 2016). The standard Tier 1 Terrain-corrected orthorectified Landsat images archived in the Google Earth Engine (GEE) platform between 1 January 1992 and 31 December 2015 were used to classify land cover in the land cover change hotspots. These datasets were already atmospherically corrected; thus, no additional preprocessing was needed.

SRTM digital elevation data is an international research effort that produces digital elevation models on a near-global scale (Farr et al., 2007). The SRTM V3 product (SRTM Plus) is provided by NASA JPL at a resolution of 1 arc-second (approximately 30 m). This dataset has undergone a void-filling process using open-source data (Advanced Spaceborne Thermal Emission and Reflection Radiometer (ASTER) Global Digital Elevation Model2 (GDEM2), Global Multi-resolution Terrain Elevation Data 2010 (GMTED2010), and USGS National Elevation Dataset (NED)), unlike other versions that contain voids or have been void-filled using commercial sources. Previous studies have shown that topography has an impact on the distribution of many land cover types, thus, the 30 m spatial resolution SRTM DEM was added to the features for classification (Feng et al., 2018). Derived DEM features, such as slope and aspect, were also incorporated.

Three annual single-type land cover datasets at a global scale with 30-m spatial resolution that have been released in recent years were collected to aggregate annual land cover. These are detailed below (Table 1). Gong et al. (2020) mapped the annual Global Artificial Impervious Area (GAIA) from 1985 to 2018 using the full archive of 30-m resolution Landsat images on the GEE platform (Gong et al., 2020). The performance of a previously developed algorithm in arid areas was improved with the use of ancillary datasets, including nighttime light data and Sentinel-1 Synthetic Aperture Radar data. The mean overall accuracy of the GAIA data for 1985, 1990, 1995, 2000, 2005, 2010, and 2015 is higher than 90%. Hansen et al.’s (2013) Global Forest Change characterized global forest extent and change based on time-series analysis of Landsat images. Annual forest loss was mapped spatially at 30-m resolution. Data from 2000 to 2015 was used to improve the forest mapping accuracy. Pekel et al.’s (2016) JRC Yearly Water Classification History describes annual distribution of surface water since 1984. It was produced by applying over 3 million scenes from Landsat 5, 7 and 8 satellites and by using expert system each pixel was judged whether it was water or non-water.

The world’s first all season training and validation sample sets for global land cover classification were reported by Li based on visual interpretation and cross checking (Li et al., 2017). Except for the location, time of image acquisition, land cover type for all four seasons for FROM-GLC land cover classification systems, the dataset includes the homogeneity of the area surrounding location of each sample, which classifies the “size” of a sample unit into 1 × 1, 3 × 3, 9 × 9, 17 × 17, and 33 × 33 pixels to mark the suitability for application on 30, 100, 250, 500, and 1,000-m scales, respectively.

In this study, we selected samples closest to growing season in the sample sets as the training samples. To be specific, we selected samples whose time of image acquisition was closest to the 183 day of the year in the Northern Hemisphere and the 365 day of the year in the Southern Hemisphere. In this way we acquired 88,941 training samples globally, including 8,776 cropland samples, 17,362 forest samples, 16,017 grassland samples, 7,817 shrubland samples, 1,701 wetland samples, 12,163 water samples, 2,570 tundra samples, 5,504 impervious surface samples and 17,031 bareland samples. Another global samples set proposed by Huang et al. (2020) has been used to check the consistency of those 88,941 training samples along years.

### Land cover change area detection

Global land cover change areas were identified by comparing existing land cover datasets. We used ESA-CCI land cover map as a primary input in this process. Initially, the classification system cross-walk was carried using the rules in Table 2. The 17 land cover types of ESA-CCI were converted into eight land cover types under the FROM-GLC classification system. Then land cover in 6 years (1992, 1995, 2000, 2005, 2010, 2015) was compared to land cover in the adjacent years under the FROM-GLC classification system. In this way, the land cover change locations of six time periods (1992 to 1995, 1995 to 2000, 2000 to 2005, 2005 to 2010, 2010 to 2015) were derived by comparing the start and the end year of that time period. The land cover change areas were used as a mask for the land cover classification in the next step.

**Table 2:** Classification schemes cross-walk strategies.

FROMGLC	ESA
Cropland	Cropland, rainfed,
	Cropland, irrigated or post-flooding,
	Mosaic cropland (>50%)/natural vegatation (tree, shrub, herbaceous cover),
	Mosaic cropland/natural vegatation (tree, shrub, herbaceous cover) (>50%)
Forest	Tree cover, broadleaved, deciduous, closed to open (>15%),
	Tree cover, needleleaved, evergreen, closed to open (>15%),
	Tree cover, needleleaved, deciduous, closed to open (>15%),
	Tree cover, mixed leaf type (broadleaved and needleleaved),
	Mosaic tree and shrub (>50%)/herbaceous cover
Grassland	Mosaic tree and shrub/herbaceous cover (>50%), Grassland,
Shrubland	Shrubland
Wetland	Tree cover, flooded fresh or brakish water,
	Tree cover, flooded, saline water,
	Shrub or herbaceous cover, flooded, fresh/saline/brakish water
Water	Water bodies
Impervious surface	Urban areas
Bareland	Sparse vegetation (tree, shrub, herbaceous cover) (<15%), Bare areas

Another input to land cover change came from urban change. We aggregated the GAIA impervious surface land cover into our final land cover products to improve the accuracy of our land cover dataset. Because the base map we used was FROM-GLC in 2017, we know that the urban area in 2017 should be larger than the previous years. Thus, we needed to extract the expanded impervious surface area and derive the previous land cover type. Because of its coarser resolution, it was possible that the ESA-CCI product may miss some of this change. On the basis of the GAIA dataset, which is a 40-year time series, we extracted the urban expansion area of six time periods (1992–1995, 1995–2000, 2000–2005, 2005–2010, and 2010–2015). Then, we combined the land cover change locations detected by ESA-CCI and the urban expansion locations detected by GAIA to give the final land cover change area.

### Land cover classification

**Figure 2:**
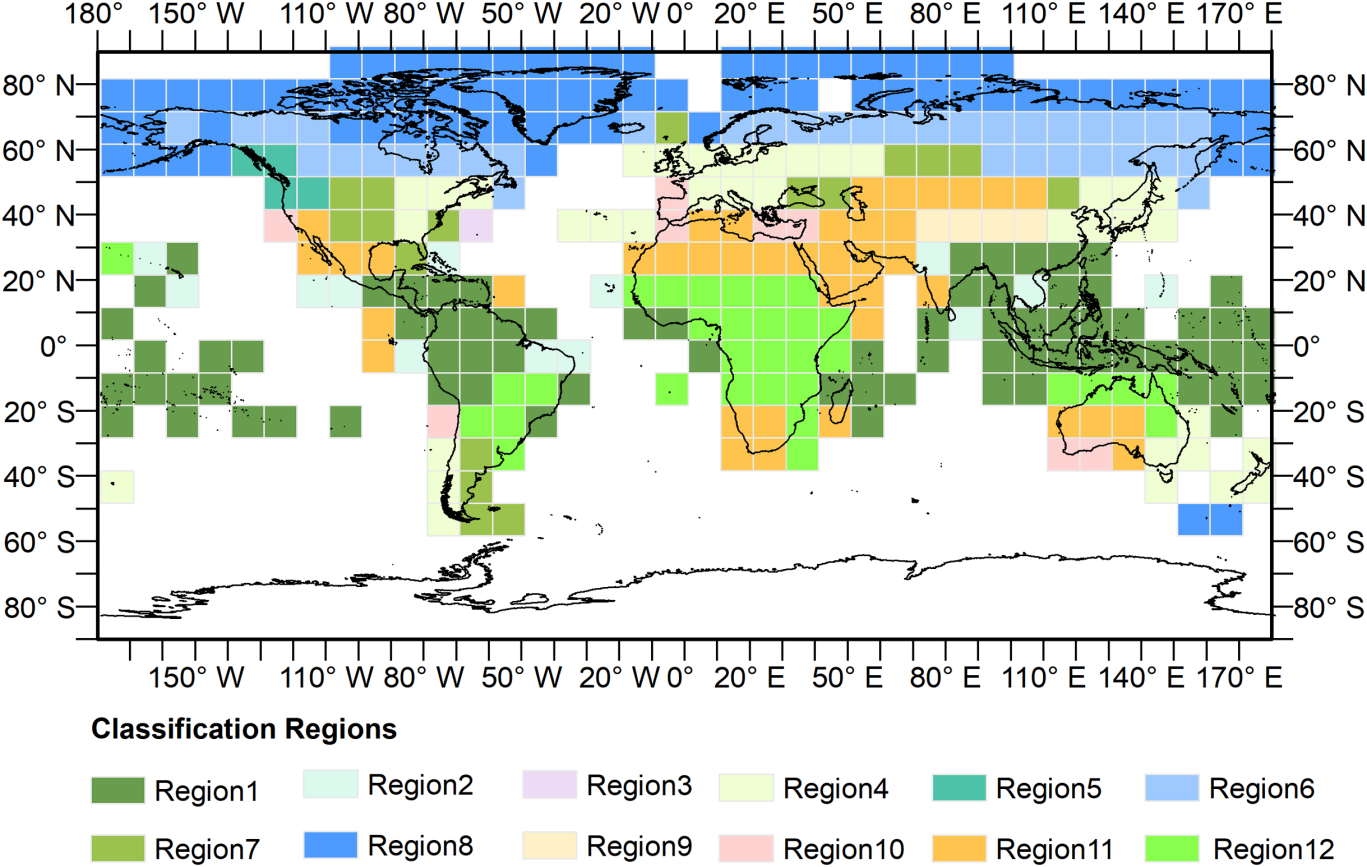
Distribution of the training regions. Training regions are represented by grids.

The GEE, consisting of a multi-petabyte analysis-ready dataset with a high-performance cloud computing platform, has enabled researchers to carry out continental and global scale spatial mapping work (Gorelick et al., 2017). The cloud-based platform presents an opportunity for researchers to monitor land cover changes rapidly and effectively over a long-time span. To build more adaptive training models for global land cover classification and avoid the issue of running out of memory owing to the calculation limitation of the GEE platform, we divided the Earth’s terrestrial area into 12 training regions to train models separately based on an ecoregions layer (Dinerstein et al., 2017). We generated a fishnet first, each grid of which was 10° × 10° in size. Only grids covering land areas were kept. Next, we calculated the area of each of 14 biomes in each grid and then we distributed the grids into different groups according to its largest area biome. The corresponding biome names of the 12 training regions was listed in Table 3 and the results for the 12 regions were shown in Fig. 2.

**Table 3:** Corresponding biome name of the 12 training regions.

Region number	Biome name
Region 1	Tropical & Subtropical Moist Broadleaf Forests
Region 2	Tropical & Subtropical Dry Broadleaf Forests
Region 3	Tropical & Subtropical Coniferous Forests
Region 4	Temperate Broadleaf & Mixed Forests
Region 5	Temperate Conifer Forests
Region 6	Boreal Forests/Taiga
Region 7	Temperate Grasslands, Savannas & Shrublands
Region 8	Tundra
Region 9	Montane Grasslands & Shrublands
Region 10	Mediterranean Forests, Woodlands & Scrub
Region 11	Deserts & Xeric Shrublands
Region 12	Tropical & Subtropical Grasslands, Savannas & Shrublands

Training samples were then filtered according to the training region boundaries. To avoid a disparity in the numbers of different land cover type samples in each classification region, we set a variable percentage for training samples in different land cover types. If the sample numbers of a particular land cover type were less than 100, samples in other regions were used to ensure the necessary sample quantity.

**Figure 3:**
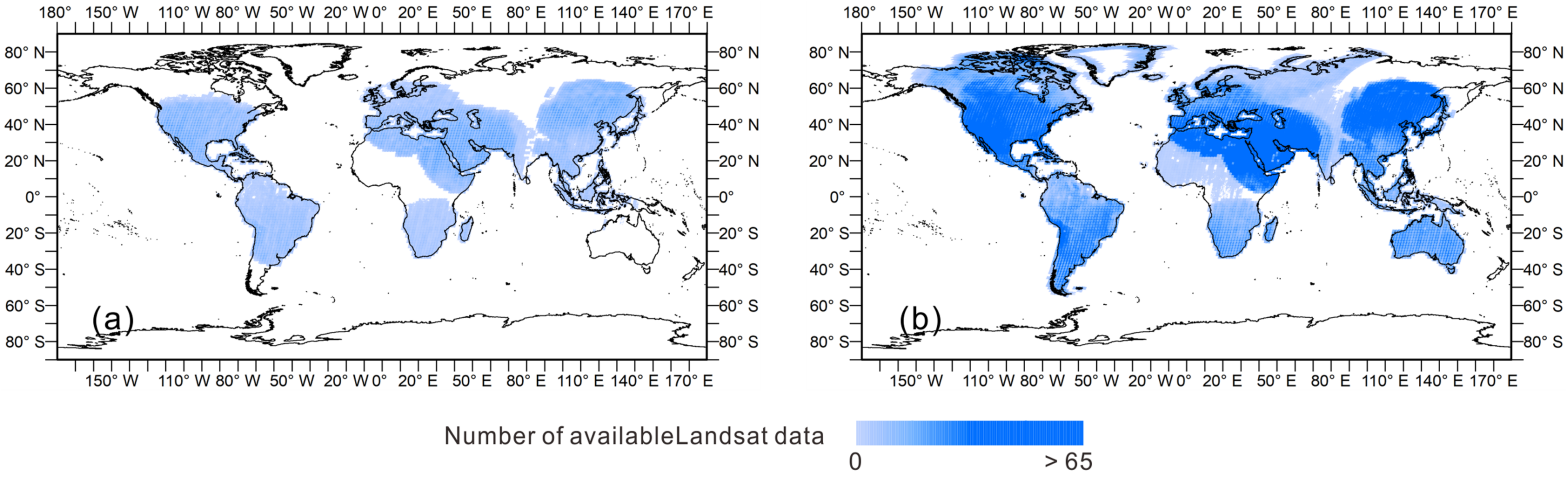
Available Landsat observations in single year and 5-year period. (A) The Landsat image availability in 2000; (B) the Landsat image availability from 1998 to 2002.

We constructed an input feature set with strong discrimination ability to detect land cover from multiple aspects, including spectra, phenology, and terrain. The percentiles (including 10, 50, 90) of all bands of Landsat images, and their derivatives including the Normalized Difference Vegetation Index (NDVI) and Modified Normalized Difference Water Index (MNDWI), were calculated. We calculated the available Landsat observations in single year (1990, 1995, 2000, 2005, 2010 and 2015) and in each 5-year period (1988–1992, 1993–1997, 1998–2002, 2003–2007, 2008–2012, and 2013–2017). Figure 3 took the available Landsat observations in 2000 as the example and that of the other years could be found in the Supplementary File. Areas were often missing in a single year of Landsat observations, mostly in the polar Arctic, middle of Africa and north of South America. To maintain the completeness of the observation and phenological information, we used the percentiles of the spectral bands and derived indices in the 5-year period as the training features.

Land cover classification was carried out on the GEE cloud computing platform. The training set and feature set were used as inputs to a Random Forest model and the number of trees was set as 100. The result identified the land cover in the land cover change locations detected in the ESA-CCI dataset in 1990, 1995, 2000, 2005, 2010 and 2015.

### Mosaic

After mapping the land cover in the land cover change locations, we generated the open synergistic land cover maps by mosaicking the classification results and the single-type land cover products with the FROM-GLC 2017 land cover map. First, we overlaid the classification results on FROM-GLC 2017 land cover map enabling the base map to vary in the 6 separate years. The forest change by Hansen provided annual forest loss from 2000 to 2019. We extracted the accumulated forest loss in four periods, including 2017–2015, 2015–2010, 2010–2005 and 2005–2000. Then, the forest loss was added to each land cover map in each corresponding year. We dealt with the GAIA and JRC Water Classification History datasets in the same way, by overlaying the two layers we extracted in the corresponding year on the previous year’s map to derive the final result. Aggregating widely accepted high quality land cover datasets has reduced the uncertainty of land cover types in our dataset including impervious, forest and water and it has also ensured the spatial and temporal consistency of these land cover types in our dataset.

## Results

### Mapping result

**Figure 4:**
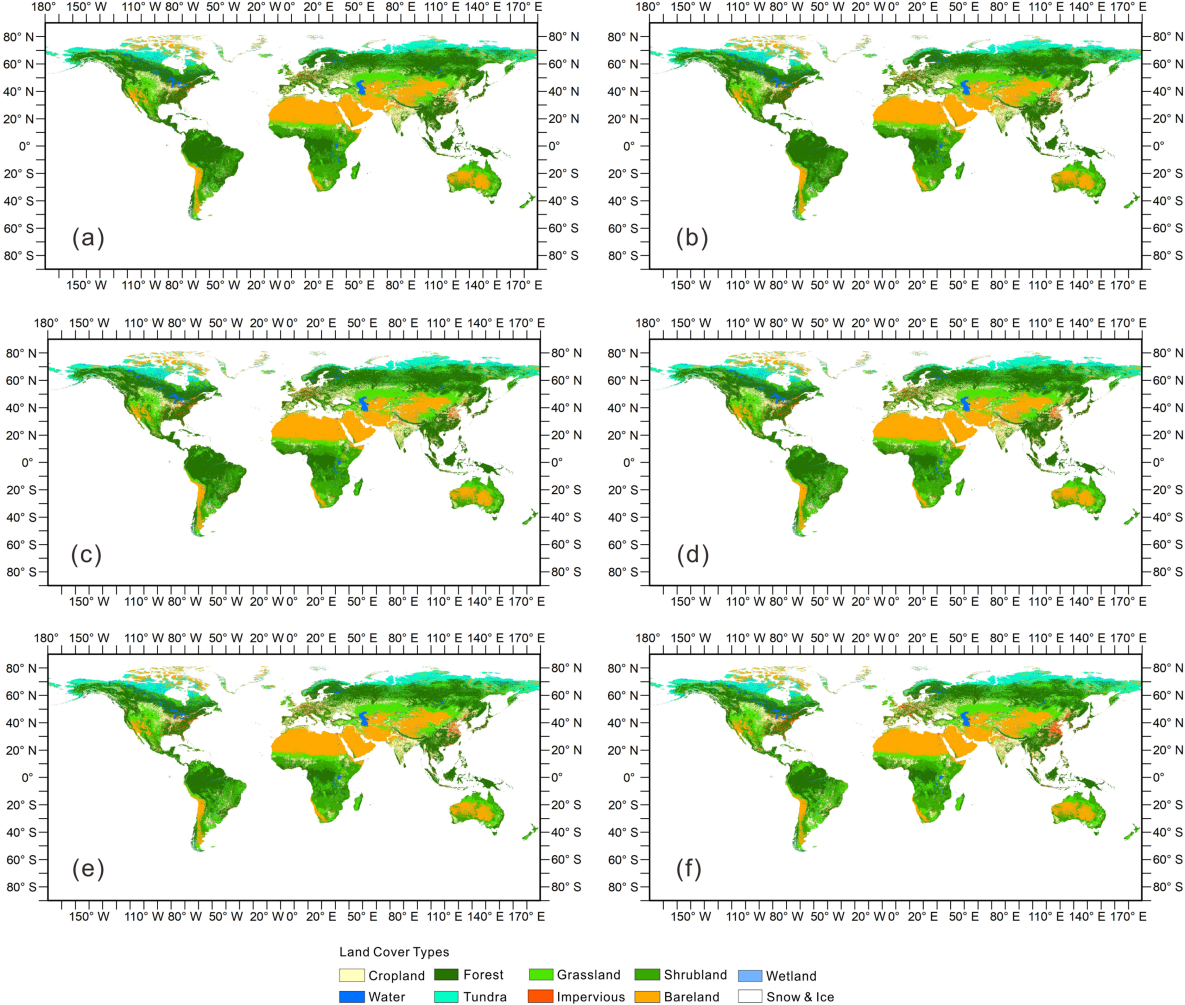
The open synergy global land cover map. (A–F) The land cover map of 1990, 1995, 2000, 2005, 2010, 2015, respectively.

On the basis of this method of classification and mosaicking, we derived a global land cover dataset for 6 years over a time span of 25 years. The result is shown in Fig. 4. This dataset can be obtained from the following collection snippet through the GEE platform: ee.ImageCollection(“users/naisoild/OpenSynergistic”). Due to the upload limit we made a land cover percentage dataset with 1-km spatial resolution which is available through Figshare: https://doi.org/10.6084/m9.figshare.14329025.v1.

**Figure 5:**
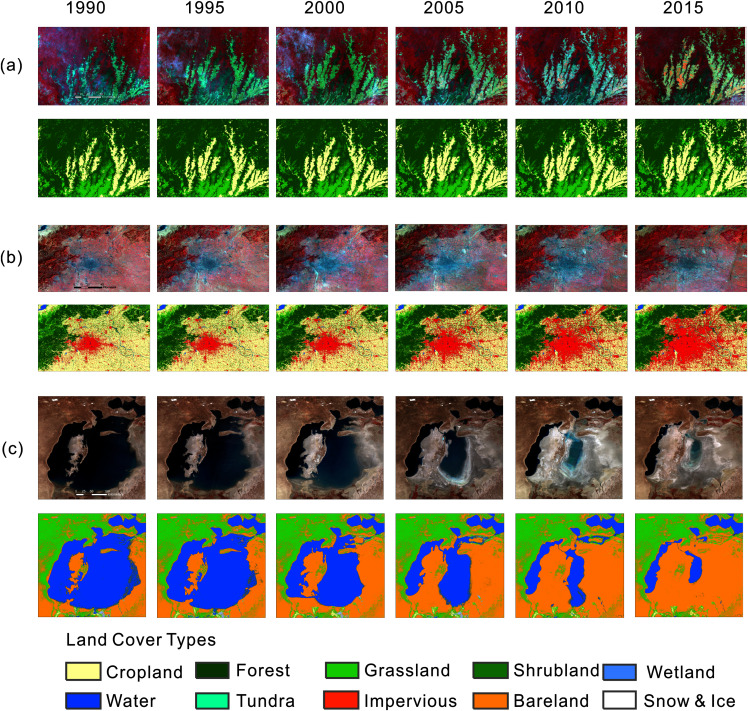
Examples of our mapping result. (A, B, C) The conversion of forest to cropland in the Amazon, the urban expansion of Beijing and the contraction of the Aral Sea.

Figure 5 shows specific examples of our mapping results. Our product effectively describes the changes in these locations, even though the type of land cover changes differed. However, our results also had several limitations. For instance, because we detected land cover changes mostly from the ESA-CCI land cover series with a resolution of 300 m and overlaid subsequent land cover classifications, the classification results tended to vary from the base map in the boundary areas, which may produce edge effects. Furthermore, our results preserved errors in the datasets that we aggregated in this study, such as the misclassification of the water area of the Aral Sea in 1995 shown in Fig. 5B.

Our method, based on the land cover change detection and classification, could produce land cover maps with up to annual frequency if annual observations are obtainable, such as Landsat 8 and Sentinel 2. Furthermore, our mapping framework is open to the input of high quality and high observation frequency local land cover datasets. Local datasets could be aggregated into our dataset through cross-walk as well.

### Technical validation

**Figure 6:**
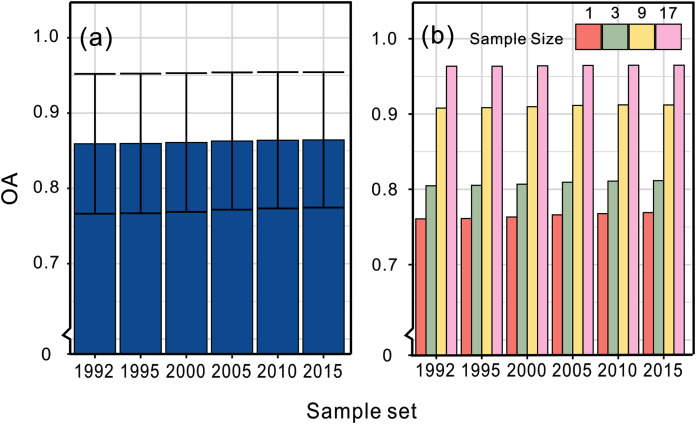
Overall accuracies of our land cover datasets. (A) The average accuracy and (B) the specific accuracy validating by sample sizes of 1, 3, 9 and 17, respectively.

The land cover maps, covering six periods, were validated by the first global all season validation sample set compiled by Li containing ~35,000 validation samples interpreted on Landsat 8 from 2013 to 2015 (Li et al., 2017). The accuracies using the above validation sample set are shown in Fig. 6. By validating the results using different sample sizes, we found that the accuracy increased with larger sample sizes indicating more homogeneity. The NLCD research team developed a suite of intermediate products including land cover change disturbance date and we evaluated the change detection accuracy of our product by this dataset in the NLCD covering area. We used the 33 samples which located in the change regions of our maps and the mapping region of NLCD from validation samples set and then we check the consistency of the change year of the two datasets. The result showed that 22 out of 33 samples had the same change period, indicating 66.7% change detection accuracy. If we adopted confidence interval of ±2 years in the validation as we did when we extracted the percentiles as training features, the accuracy could be up to 87.9%.

**Figure 7:**
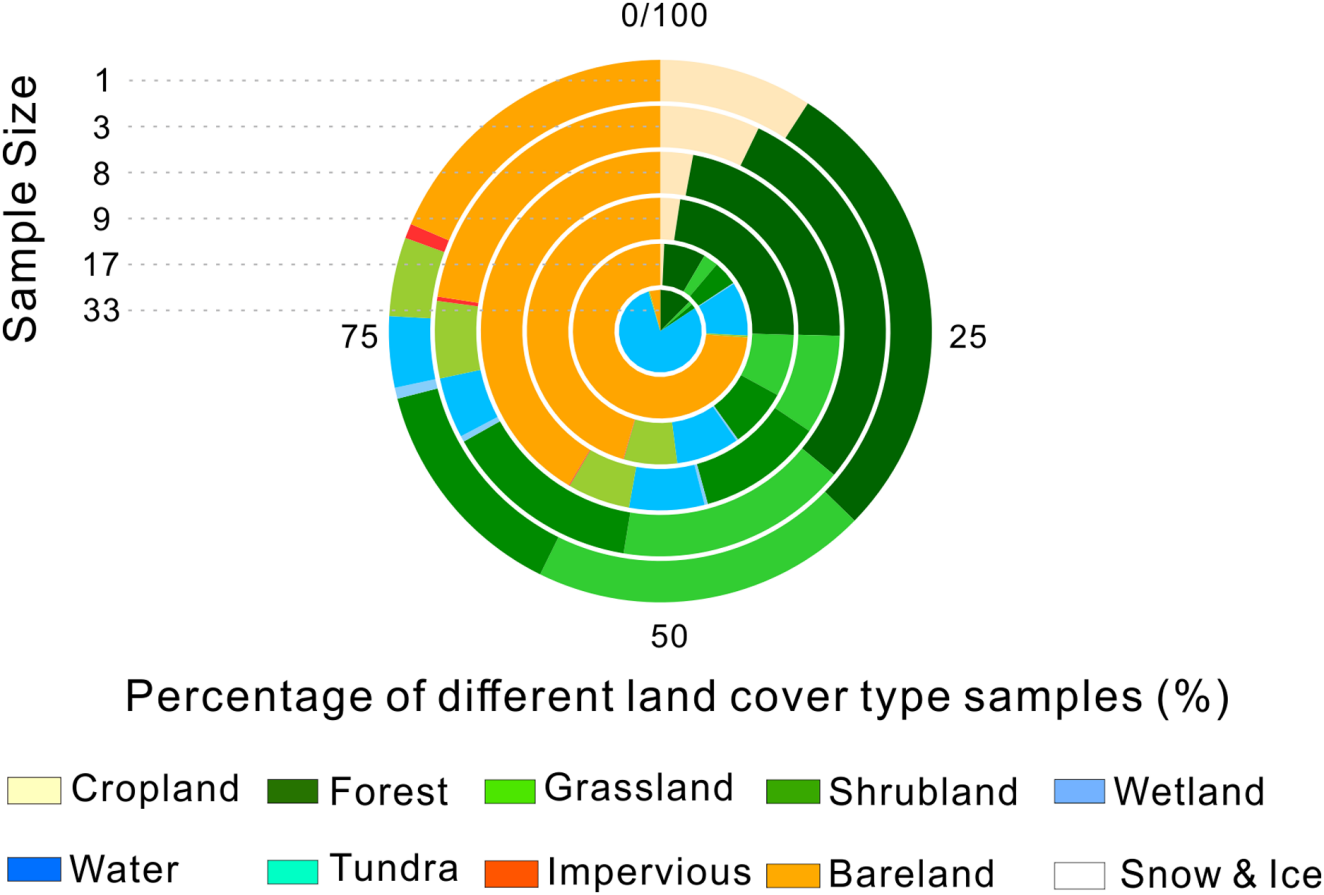
Validation sample types distribution with different sample sizes.

We calculated the percentage of each land cover in each of the validation sample sets, which is shown in Fig. 7. We found that the percentage of cropland and impervious surface decreased rapidly as the sample size increased. At the same time, homogenous land cover types such as bareland and water occupied a higher proportion. When the sample size was set as 33, bareland and water made up nearly all of the validation samples. That could explain why accuracy increased when validation samples with larger sample size were used. This suggests that extra attention should be paid when samples with large sample size are used for validatation because of their potential distribution bias.

**Figure 8:**
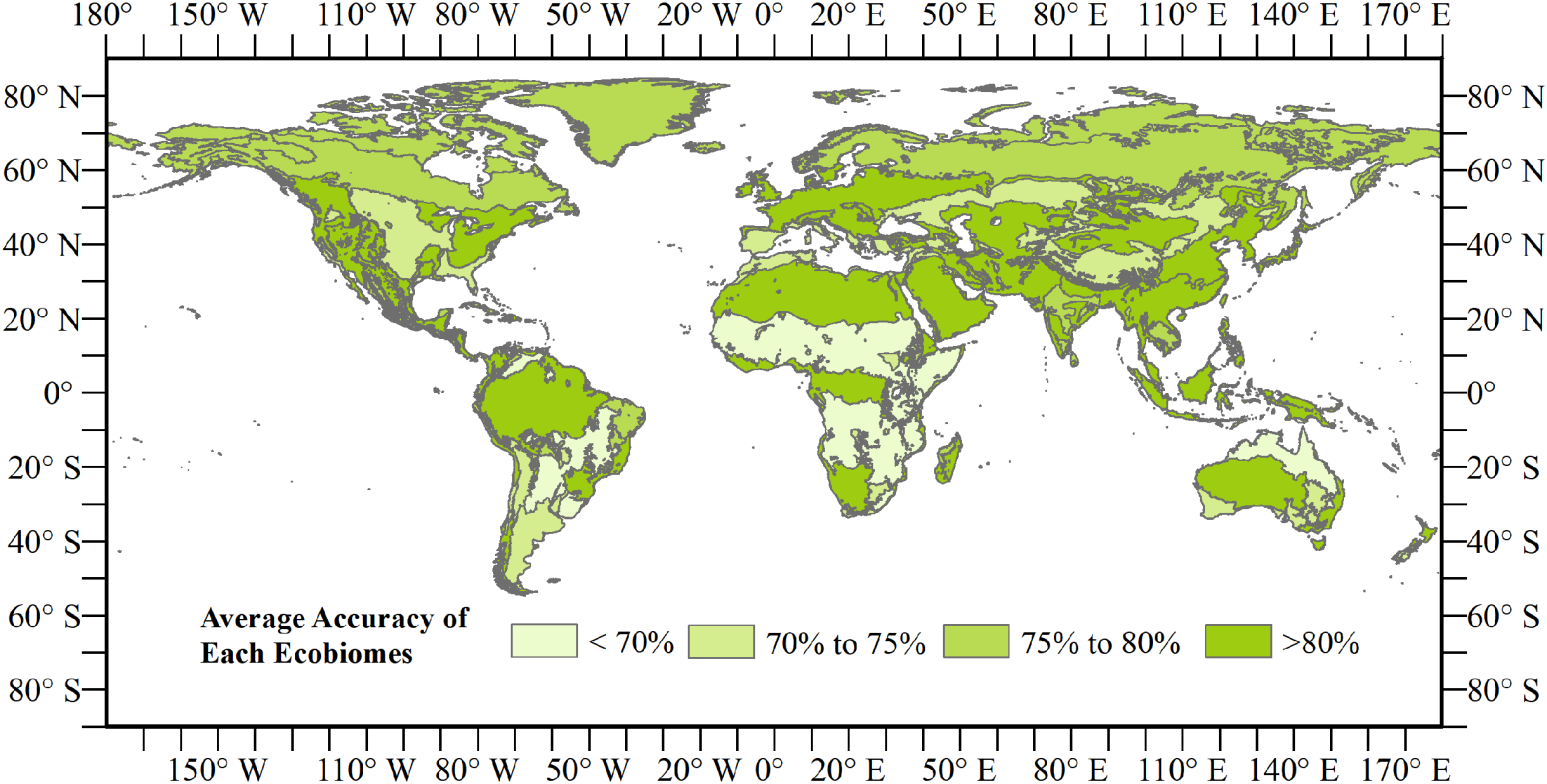
Overall accuracy of the land cover map for 2015 in each ecoregion.

We calculated the accuracy of the land cover map of 2015 in each ecoregion using the validation samples of Li to check the classification heterogeneity (Fig. 8). Here, an ecoregion layer was used to filter the validation samples in each of the 847 ecoregions and calculate the accuracies of 774 ecoregions with samples fell in Dinerstein et al. (2017). The results showed that most ecoregions reached accuracies of between 70% and 75%. Some ecoregions had an overall accuracy more than 75%, such as Siberia and northern Sahara, which has uniform land cover types. Few ecoregions had accuracies lower than 70%.

## Discussion

**Figure 9:**
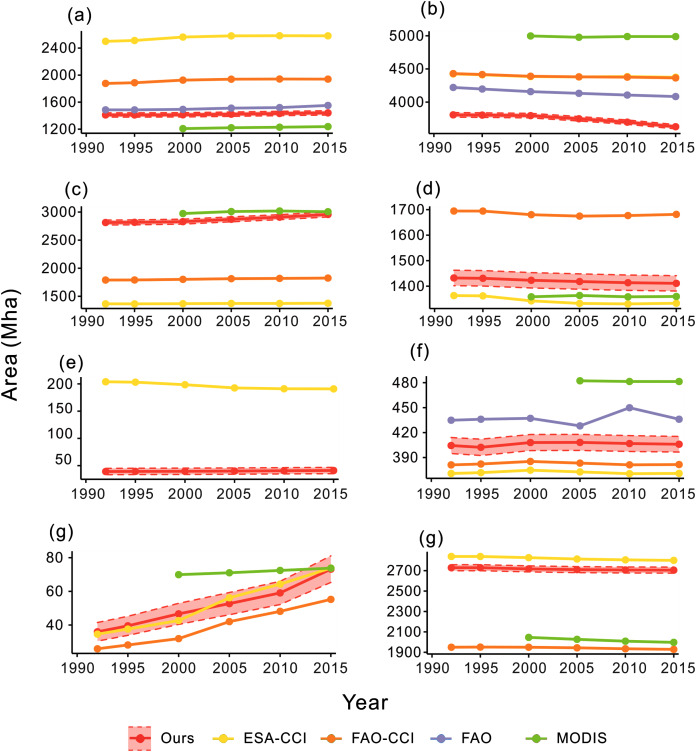
Area curves of global land cover change every 5 years from 1992 to 2015. (A–G) The land cover change of cropland, forest, grassland, shrubland, wetland, water, impervious surface and bareland, respectively.

We compared the calculated area of our product with statistics of the Food and Agriculture Organization of the United Nations (FAO) and other classification results including ESA-CCI, FAO_CCI_LC and FAO_MODIS. The results are shown in Fig. 9. We first compared the change of cropland from our open synergistic global land cover dataset with the cropland area from the FAO statistics and other mapping datasets. Cropland in our dataset increased since 1992, showing the same trend as the other datasets. The FAO statistics had the most similar area to our dataset, indicating that the cropland area of our dataset was relatively accurate. Forest decreased in all the datasets, although forest in our dataset was less than other datasets. Our result showed that grassland increased over the last 20-year period. The FAO-MODIS result was the closest to our dataset, while the ESA-CCI and FAO_CCI_LC had less grassland compared with our dataset. Shrubland showed a decreasing trend in all land cover datasets from 1992 to 2000 except for FAO_MODIS because of the data availability. Only the ESA-CCI and our result had wetland land cover. The results varied and were different to the FROM-GLC 2017 result. Wetland in ESA-CCI product decreased from 1992 to 2000 but the change was not obvious in our product. The water class of our product mainly came from the JRC yearly history. The water area of the five datasets was close and the area of our dataset was in the middle of the five datasets. The area of impervious surfaces increased in all the four datasets. The ESA-CCI was the closest dataset to our results. For bareland, our result was close to the ESA-CCI result. Two datasets provided by FAO were close to, but lower than, our result.

## Conclusions

In this study we generated an open synergistic land cover dataset for 6 years—1990, 1995, 2000, 2005, 2010 and 2015. The overall accuracies of the six maps were around 75% and the accuracy for change area detection was over 70%. Our product also showed good similarity with the FAO and existing land cover maps. The classification system included seven classes: cropland, forest, grassland, shrubland, wetland, water, tundra, impervious surface and bare land. The dataset was presented with an image collection divided into 10° × 10° squares, which is convenient for readers to filter their region of interest or mosaic into a whole image.

## Supplemental Information

10.7717/peerj.11877/supp-1 Supplemental Information 1 Available Landsat observations in single year and 5-year period. (a), (c), (e), (g) and (i) exhibit the Landsat image availability in single year of 1990, 1995, 2005, 2010 and 2015; (b), (d), (f), (h) and (j) exhibit Landsat image availability in 5-year period of 1988–1992, 1993–1997, 2003–2007, 2008–2012 and 2013–2017.

## Additional Information and Declarations

### Data Availability

The following information was supplied regarding data availability: The land cover percentage dataset with 1-km spatial resolution is available at figshare: Zhao, Jiyao; Yu, Le; Liu, Han; Huang, Huabing; Wang, Jie; Gong, Peng (2021): Open Synergy Land Cover Series. figshare. Dataset. DOI 10.6084/m9.figshare.14329025.v1.
