# Supplementary figures and images for: Towards an open and synergistic framework for mapping global land cover
Source: PeerJ. 2021 Aug 4;9:e11877. doi: 10.7717/peerj.11877 (PMC8349160; doi:10.7717/peerj.11877)

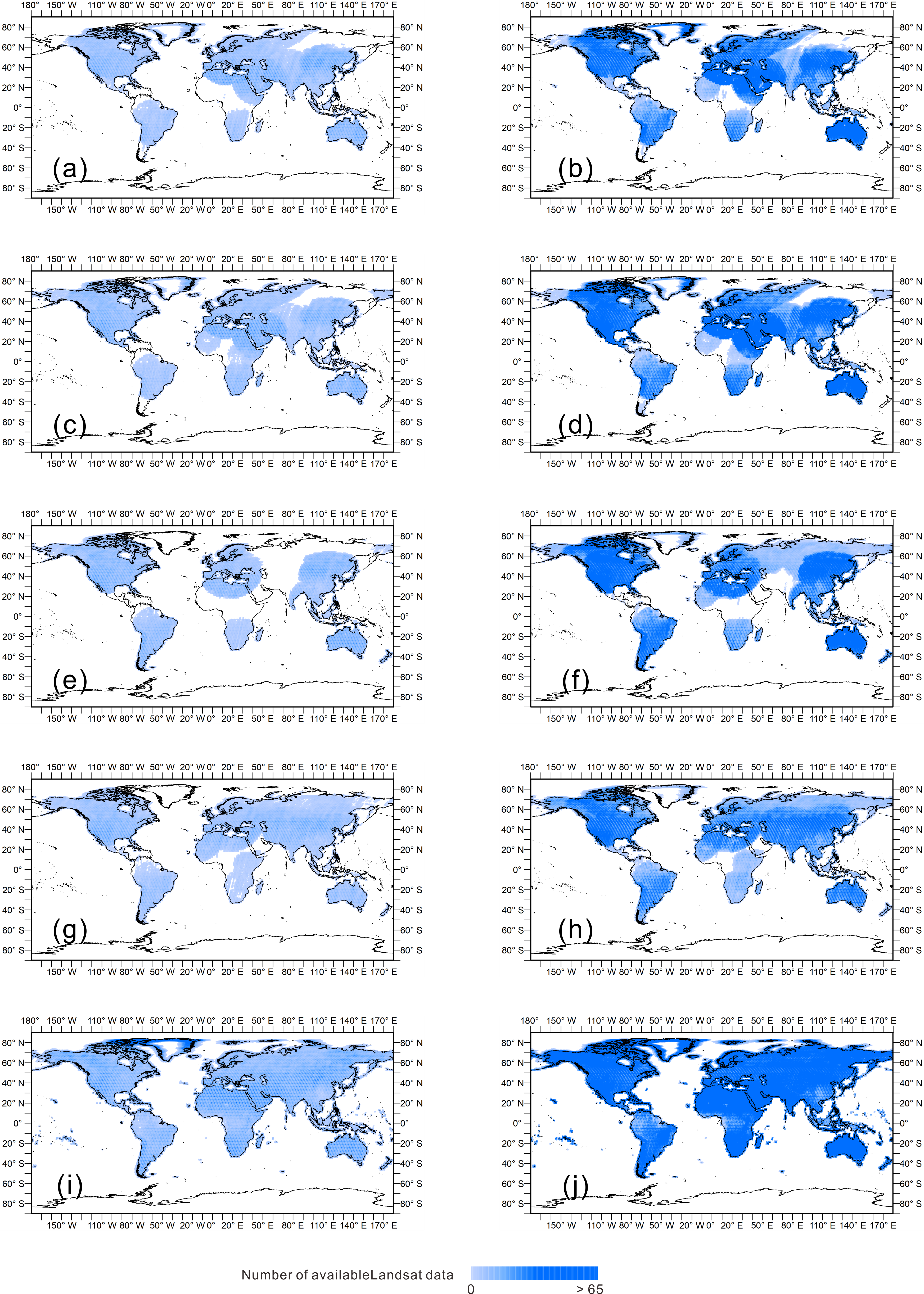

Supplement: Supplemental Information 1 — (a), (c), (e), (g) and (i) exhibit the Landsat image availability in single year of 1990, 1995, 2005, 2010 and 2015; (b), (d), (f), (h) and (j) exhibit Landsat image availability in 5-year period of 1988–1992, 1993–1997, 2003–2007, 2008–2012 and 2013–2017. [file peerj-09-11877-s001.png]
